# Strawberry-Derived Exosome-Like Nanoparticles Prevent Oxidative Stress in Human Mesenchymal Stromal Cells

**DOI:** 10.3390/biom11010087

**Published:** 2021-01-12

**Authors:** Francesca Perut, Laura Roncuzzi, Sofia Avnet, Annamaria Massa, Nicoletta Zini, Silvia Sabbadini, Francesca Giampieri, Bruno Mezzetti, Nicola Baldini

**Affiliations:** 1 BST Biomedical Sciences and Technologies Lab, IRCCS Istituto Ortopedico Rizzoli, 40136 Bologna, Italy; francesca.perut@ior.it (F.P.); laura.roncuzzi@ior.it (L.R.); sofia.avnet@ior.it (S.A.); annamaria.massa@ircc.it (A.M.); 2 CNR Institute of Molecular Genetics “Luigi Luca Cavalli-Sforza”, Unit of Bologna, 40100 Bologna, Italy; nicoletta.zini@cnr.it; 3 IRCCS Istituto Ortopedico Rizzoli, 40136 Bologna, Italy; 4 Department of Agricultural, Food and Environmental Sciences, Università Politecnica delle Marche, 60121 Ancona, Italy; s.sabbadini@staff.univpm.it (S.S.); b.mezzetti@staff.univpm.it (B.M.); 5 Department of Clinical Specialistic and Odontostomatological Sciences, University Politecnica delle Marche, 60121 Ancona, Italy; f.giampieri@staff.univpm.it; 6 Department of Biochemistry, Faculty of Sciences, King Abdulaziz University, Jeddah 21589, Saudi Arabia; 7 College of Food Science and Technology, Northwest University, Xi’an 710069, China; 8 Department of Biomedical and Neuromotor Sciences, University of Bologna, 40126 Bologna, Italy

**Keywords:** extracellular vesicles (EVs), edible plant-derived exosome-like nanoparticles (EPDENs), *Fragaria* x *ananassa*, miRNA, ascorbic acid, oxidative stress, mesenchymal stromal cells

## Abstract

Plant-derived exosome-like nanovesicles (EPDENs) have recently been isolated and evaluated as potential bioactive nutraceutical biomolecules. It has been hypothesized that EPDENs may exert their activity on mammalian cells through their specific cargo. In this study, we isolated and purified EPDENs from the strawberry juice of *Fragaria* x *ananassa* (cv. Romina), a new cultivar characterized by a high content of anthocyanins, folic acid, flavonols, and vitamin C and an elevated antioxidant capacity. *Fragaria*-derived EPDENs were purified by a series of centrifugation and filtration steps. EPDENs showed size and morphology similar to mammalian extracellular nanovesicles. The internalization of *Fragaria*-derived EPDENs by human mesenchymal stromal cells (MSCs) did not negatively affect their viability, and the pretreatment of MSCs with *Fragaria*-derived EPDENs prevented oxidative stress in a dose-dependent manner. This is possibly due to the presence of vitamin C inside the nanovesicle membrane. The analysis of EPDEN cargo also revealed the presence of small RNAs and miRNAs. These findings suggest that *Fragaria*-derived EPDENs may be considered nanoshuttles contained in food, with potential health-promoting activity.

## 1. Introduction

Extracellular vesicles (EVs) are heterogeneous, nanosized vesicles that are constitutively released by almost all types of eukaryotic and prokaryotic cells [1,2]. They contain metabolites, proteins, lipids, RNAs, miRNAs, mRNAs, and DNAs and can transfer their cargo to recipient cells, playing a fundamental role as extracellular messengers to mediate cell–cell communication [3]. The critical role played by EVs in the mediation of cell–cell communication has been identified both in health and disease and in bacterial, fungal, and animal kingdoms [4,5,6,7,8]. An increasing number of studies have shown the existence of plant-derived nanosized particles, the characteristics of which resemble those of mammalian exosomes [9,10,11,12]. Exosome-like nanoparticles (ELNs) may have originally evolved in plants as a means of communication between plant cells and as a way of modulating the first-line innate immune defenses that plants deploy upon pathogen invasion [13]. The release of ELNs has recently become a subject of increasing interest regarding the possibility of intercellular communication, even between different species [7]. In this context, increasing evidence suggests that edible plant-derived exosome-like nanovesicles (EPDENs) can be absorbed in the mammalian gastrointestinal tract and have the potential to mediate plant–animal intercellular communication [13,14,15,16,17]. Mu et al. demonstrated that nanoparticles from edible plants (grape, grapefruit, ginger, and carrot) have anti-inflammatory properties and help to maintain intestinal homeostasis [10]. Moreover, ginger-derived nanoparticles can protect against the development of liver-related diseases such as alcohol-induced damage [16]. We recently demonstrated that EPDENs from *Citrus limon* L. contain small RNA, vitamin C, and citrate; this cargo is able to prevent oxidative stress in human cells and modulate their differentiation versus the osteogenic lineage [12]. The potential health benefit associated with dietary intake of fruit has attracted increasing interest. Among berries, strawberry is a rich source of several nutritive and non-nutritive bioactive compounds, which are implicated in various health-promoting and disease-preventive measures [18].

Strawberries are widely cultivated all over the world, with high appreciation by consumers [19]. Among the different strawberry cultivars available on the market, Romina, released in 2011, is a new cultivar and extensively studied to determine the compositional and nutritional characteristics of the fruit [20]. This cultivar presents high adaptability to nonfumigated soil and resistance to the major strawberry diseases. It is of interest to producers and consumers due to its early ripening time and nutritional quality. Romina fruit is acclaimed for its higher content of soluble solids combined with low total acidity, which confer the fruit with a very high perception of sweetness [20]. Romina fruit also combines a high content of anthocyanins, folic acid, flavonols, and vitamin C and an elevated antioxidant capacity [20,21].

In this study, for the first time, we isolated, purified, and characterized exosome-like nanoparticles from the strawberry juice of *Fragaria* x *ananassa* (cv. Romina). We demonstrated that *Fragaria*-derived nanovesicles possess a similar size and structure to mammalian-derived exosomes. *Fragaria-*derived EPDENs were taken up and internalized by human mesenchymal stromal cells and did not exert cytotoxic effects on the cells. Moreover, *Fragaria*-derived EPDENs prevented oxidative stress in human cells. Analysis of exosomal cargo revealed the presence of small RNAs, miRNAs, and a high content of vitamin C. The potential impact of these findings on the progress toward health benefits and food-derived technology is discussed.

## 2. Materials and Methods 

### 2.1. Fruits Harvest and Sampling 

Strawberry fruits (*Fragaria* x *ananassa*) of cultivar Romina were picked from plants grown in the P. Rosati University experimental farm, Agugliano (Ancona, Italy), in open-field conditions, according to the plastic hill culture production system. Strawberries for analysis were harvested fully red, at the second, third, and fourth main pickings, and stored at −80 °C until analyzed.

### 2.2. Cell Culture 

Adipose-derived mesenchymal stem cells (ADMSCs) were purchased from the American Type Culture Collection (Milan, Italy). Cells were grown in α-minimum essential medium (α-MEM) supplemented with 10% heat-inactivated fetal bovine serum (FBS), 100 U/mL penicillin, and 0.1 mg/mL streptomycin (Sigma-Aldrich, Milan, Italy). Cells were maintained at 37 °C in a humidified 5% CO_2_ atmosphere. Passage 2–3 ADMSCs were used in all experiments. FBS depleted of exosomes (FDE) was obtained via ultracentrifugation at 110,000× *g* overnight at 4 °C (Beckman Coulter, Milan, Italy). FDE was used for all cell culture experiments.

### 2.3. Isolation and Purification of Fragaria-Derived EPDENs

Strawberries (500 g) were washed with water, squeezed using a potato masher (Tescoma, Brescia, Italy), and then strained with a colander. The collected juice (250 mL) was differentially centrifuged at 3000× *g* (for 30 min) and 10,000× *g* (for 1 h) at 4 °C. The supernatant was filtered using a cell strainer (100 mesh) and centrifuged at 16,500× *g* for 1 h at 4 °C three times. The supernatant was then filtered through a 0.45 µm pore-size filter and ultracentrifuged at 110,000× *g* for 1 h at 4 °C. The EPDEN pellet was washed with phosphate-buffered saline (PBS) and ultracentrifuged at 110,000× *g* for 1 h at 4 °C. The EPDEN pellet was resuspended in sterile PBS and stored at −80 °C until use. EPDEN quantity was determined by the Bradford method (Bio-Rad, Milan, Italy).

### 2.4. Isolation and Purification of Citrus limon L.-Derived EPDENs

*Citrus limon* L. (500 g) was washed in cold water and squeezed using a Citrus sprayer device (Tescoma), and 150–200 mL of juice was obtained. EPDENs were isolated as previously described [12]. Briefly, the collected juice was differentially centrifuged at 3000× *g* (for 30 min) and 10,000× *g* (for 1 h) at 4 °C. The supernatant was filtered by using 1.6 µm and 0.45 µm filters and centrifuged at 16,500× *g* for 3 h at 4 °C. The supernatant was ultracentrifuged at 110,000× *g* for 1 h at 4 °C. The EPDEN pellet was washed in cold PBS and ultracentrifuged at 110,000× *g* for 1 h at 4 °C. The EPDEN pellet was resuspended in sterile PBS and stored at −80 °C until use. 

### 2.5. Isolation and Purification of ADMSC-Derived EVs

ADMSCs were cultured until 70% confluence. Cells were washed with PBS and incubated for 48 h with serum-free α-MEM (Sigma-Aldrich). The supernatant was collected after two consecutive periods (18 h and an additional 18 h). The EVs were purified by differential centrifugation: 500× *g* for 10 min (two times), 2000× *g* for 15 min (two times), and 10,000× *g* for 30 min (two times) at 4 °C. The supernatant was then ultracentrifuged at 110,000× *g* for 1 h at 4 °C. The EV pellet was resuspended in PBS and centrifuged at 110,000× *g* for 1 h at 4 °C. The EV pellet was resuspended in sterile PBS and stored at −80 °C until use. 

### 2.6. Transmission Electron Microscopy (TEM)

*Fragaria*-derived EPDENs, *Citrus limon L.*-derived EPDENs, and ADMSC-derived EVs were resuspended in 2% paraformaldehyde (PFA) and loaded onto formvar carbon-coated grids (Electron Microscopy Sciences, Hatfield, PA, USA). Subsequently, the EPDENs and EVs were fixed in 1% glutaraldehyde, washed, and contrasted with a solution of uranyl oxalate (pH 7) embedded in a mixture of 4% uranyl acetate and 2% methylcellulose before observation with a Zeiss-EM 109 electron microscope (Zeiss, Oberkochen, Germany). The diameter of EPDENs and EVs was measured, and the percentage of size distribution was calculated.

### 2.7. Fragaria-Derived EPDENs Labeling and Uptake

*Fragaria*-derived EPDENs were stained with PKH26 Red Fluorescent Cell Linker Kit for General Cell Membrane Labeling (Sigma-Aldrich), according to the manufacturer’s instructions, with minor modifications, as previously described [22]. Unincorporated dye from EPDEN labeling reactions was removed using Exosome Spin Columns (MW 3000) (Thermo Fisher Scientific, Waltham, MA, USA), according to the manufacturer’s instructions. PKH26-labeled EPDENs (2 µg/L × 10^4^ cells), or the same volume of the PKH26-PBS control, were added to semiconfluent ADMSCs. After 4 h and 24 h of incubation, uptake was stopped by washing and fixation in 3.7% PFA for 10 min. Cells were then stained with a FITC-conjugated phalloidin (Sigma-Aldrich) and visualized with a Nikon Eclipse E800M fluorescence microscope (Nikon, Tokyo, Japan).

### 2.8. Cell Viability Assay

To evaluate the possible cytotoxic effect of *Fragaria*-derived EPDENs, ADMSCs were treated with nanovesicles at different concentrations (0–2–4–9 µg/1 × 10^4^ cells) for 48 h and 120 h. ADMSCs were harvested, and the number of viable cells was evaluated by the erythrosine B (Sigma-Aldrich) dye vital staining. All experiments were performed two times in triplicate.

### 2.9. L-Ascorbic Acid Detection

*Fragaria*-derived EPDENs were treated with protease inhibitors (Roche Diagnostics, Monza, Italy) and sonicated in a sonicator bath (Bandelin Electronic GmbH & Co. KG, Berlin, Germany) in the dark for a very short time. *Fragaria*-derived EPDENs and juice were centrifuged at 13,000× *g* for 5 min at 4 °C to remove membrane debris. Ascorbate (L-ascorbic acid or vitamin C) concentration was quantified using the ascorbate assay kit (Cayman Chemical, Ann Arbor, Michigan, USA). Fluorescence was read utilizing a microplate reader set to an excitation wavelength of 360 nm and an emission wavelength of 465 nm (Tecan Infinite F200pro, Milan, Italy). The experiments were performed three times in duplicate.

### 2.10. Determination of Antioxidant Activity

ADMSCs were pretreated with *Fragaria*-derived EPDENs (0.5–1–2 μg/mL) for 24 h followed by 400 µM hydrogen peroxide (H_2_O_2_) exposure for 24 h. The H_2_O_2_ concentration that caused the inhibition of 50% cell viability (IC50) was determined from the dose–response curve ranging from 0 to 1000 µM H_2_O_2_. Cell viability was evaluated using the acid phosphatase assay (Sigma-Aldrich), as previously described [12]. Optical density was read at 405 nm using a microplate reader. Data were reported as cell survival with respect to untreated cells (set = 100%).

ROS were detected by the 2′,7′-dichlorofluorescin (DCFH) method. Briefly, ADMSCs were pretreated with EPDENs (0.5 µg/mL) for 24 h followed by 600 μM H_2_O_2_ exposure for 1 h. The H_2_O_2_ concentration that significantly increased ROS production was determined from a dose–response curve ranging from 0 to 800 µM H_2_O_2_. ADMSCs were washed and then incubated with 10 μM 5- and 6-carboxy-2′,7′-dichlorodihydrofluorescein diacetate (CM-H2DCFDA; Thermo Fisher Scientific, Monza, Italy) for 5 min at 37 °C. The fluorescent signal of DCF obtained from the conversion of 2′,7′-dichlorofluorescin-diacetate (DCFH-DA) by the intracellular ROS produced was read at 485–535 nm using a microplate reader (Tecan Infinite F200pro). The results were expressed as the mean of the relative fluorescence units (RFUs).

### 2.11. RNA Sequencing Analysis

Total RNA was extracted from *Fragaria*-derived EPDENs and *Fragaria* juice using the mirVana™ miRNA Isolation Kit (Thermo Fisher Scientific), according to the manufacturer’s protocol. Plant RNA Isolation Aid (Thermo Fisher Scientific) was used to remove common plant contaminants such as polyphenols and polysaccharides. RNA-seq analysis was performed by IGA Technology Services (Udine, Italy). The TruSeq Small RNA Sample Prep kit (Illumina, San Diego, CA, USA) was used for library preparation, following the manufacturer’s instructions. Both RNA samples and final libraries were quantified using the Qubit 2.0 Fluorometer (Invitrogen, Carlsbad, CA, USA) and quality tested by the Agilent 2100 Bioanalyzer RNA Nano assay (Agilent Technologies, Santa Clara, CA, USA). Libraries were then processed with Illumina cBot for cluster generation on the flow cell, following the manufacturer’s instructions, and sequenced on single-end mode at the multiplexing level requested on HiSeq2500 (Illumina, San Diego, CA, USA). The CASAVA 1.8.2 version (Illumina) of the Illumina pipeline was used to process raw data for both format conversion and de-multiplexing. To identify specific miRNA, the miRBase database was used (www.mirbase.org).

### 2.12. Statistical Analysis

Statistical analysis was performed by the Stat View™ 5.0.1 software (SAS Institute, Cary, NC, USA). Quantitative results were expressed as arithmetic mean plus or minus the standard deviation (SD). The Mann–Whitney U test was used in a paired analysis, and *p* < 0.05 was considered statistically significant.

## 3. Results

### 3.1. Identification and Characterization of EPDENs from Fragaria x Ananassa

**Figure 1:**
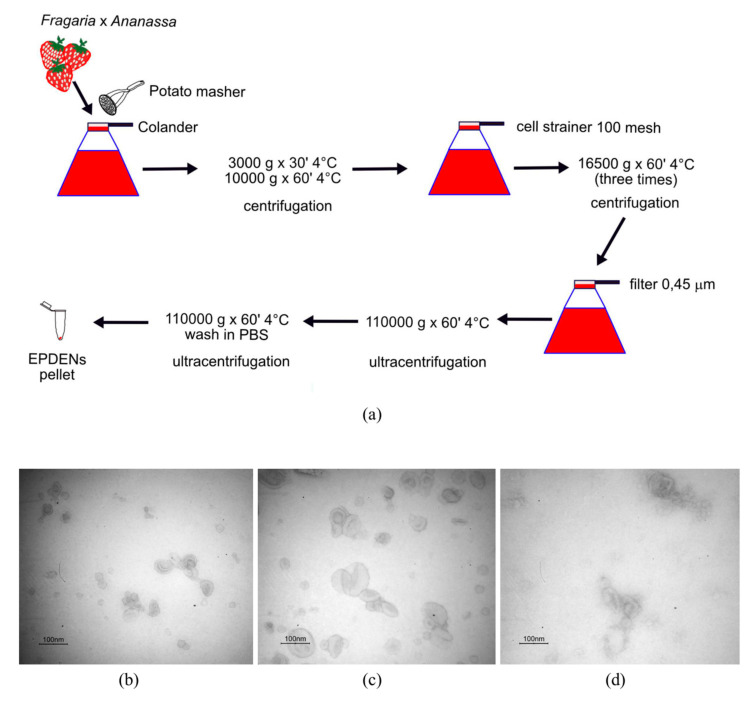
Isolation and characterization of *Fragaria*-derived exosome-like nanovesicles (EPDENs). (**a**) Schematic representation of the method used to isolate and purify EPDENs from *Fragaria* x *ananassa*. Transmission electron microscopy analysis of nanovesicles isolated from (**b**) *Fragaria* x *ananassa*, (**c**) *Citrus limon* L., and (**d**) adipose-derived mesenchymal stem cells (ADMSCs) (scale bar: 100 nm).

EPDENs were isolated from *Fragaria* x *ananassa* (cv. Romina) juice through a series of differential centrifugation, ultracentrifugation, and filtration steps (Figure 1a). From 250 mL of juice, a yield of 18 ± 3 µg nanovesicles was obtained, and based on their characteristic round- or cup-shaped morphology and dimension, these were identified as exosome-like nanovesicles. Indeed, transmission electron microscopy analysis showed that the nanovesicles isolated from *Fragaria* were morphologically homogeneous, ranging from 30 to 191 nm in size, with a typical round- or cup-shaped appearance similar to those of EVs from mammalian cells or EV-derived plant cells (*Citrus limon* L. juice) (Figure 1b–d). The size distribution of EPDENs and EVs is described in Table 1.

### 3.2. Uptake of Fragaria-Derived EPDENs

**Figure 2:**
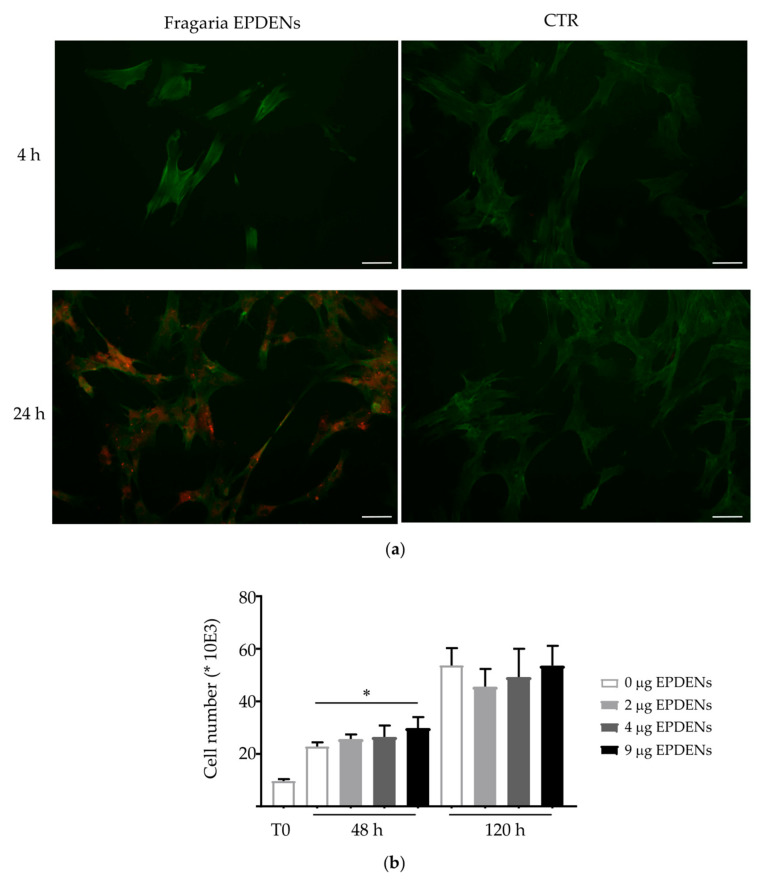
Effects of *Fragaria*-derived EPDENs on ADMSCs. (**a**) Uptake of *Fragaria*-derived EPDENs by ADMSCs. The uptake of the fluorescently labeled EPDENs (red) was evident in ADMSCs after 24 h of incubation. No stain was revealed in the untreated cells (CTR). Actin filaments were stained with a FITC-conjugated phalloidin (green). Scale bar: 10 µm. (**b**) Effect of *Fragaria*-derived EPDENs on ADMSC viability. Cells were treated with the indicated concentration of EPDENs for 48 h and 120 h. Data are reported as the cell number (mean ± SD, * *p* < 0.05).

To examine whether *Fragaria*-derived EPDENs could be taken up by mammalian cells, PKH26 labeled nanovesicles were incubated with ADMSCs at different time points and examined using fluorescence microscopy. After 24 h incubation, as shown in Figure 2a, *Fragaria*-derived EPDENs were taken up by ADMSCs and accumulated mainly in the cytoplasm. No fluorescent signal was detected in the control (PBS).

### 3.3. Effect of Fragaria-Derived EPDENs on Cells Viability

Since ADMSCs were shown to internalize *Fragaria*-derived EPDENs, we attempted to determine whether these nanovesicles have an effect on human cells. Cell viability was examined in ADMSCs in the presence of increasing concentrations of EPDENs (0–2–4–9 µg/mL) for 48 h and 120 h. The results showed that *Fragaria*-derived EPDENs do not exert any significant toxicity on ADMSCs. Notably, ADMSCs exposed to 9 µg of EPDENs for 48 h showed a significant increase (*p* = 0.0286) in viability when compared to control cells (Figure 2b).

### 3.4. Fragaria-Derived EPDENs Contain Vitamin C

Strawberry fruit represents one of the most important sources of micronutrients, including vitamin C, which is heavily concentrated in the juice of *Fragaria* fruit. On this basis, we assessed whether vitamin C is also present in *Fragaria*-derived EPDENs. Our results revealed that *Fragaria*-derived nanovesicles contain detectable levels of vitamin C (416 nmoles/mg EVs). The *Fragaria* whole juice from which they were derived contains 1.990 µM vitamin C.

### 3.5. Antioxidant Activity of Fragaria-Derived EPDENs

**Figure 3:**
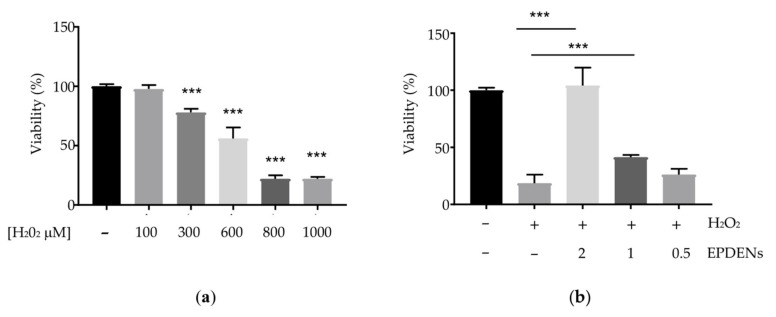
Effect of *Fragaria-*derived EPDENs on oxidative stress in human ADMSCs. (**a**) Dose-dependent effect of H_2_O_2_ on cell viability (*n* = 3) (*** *p* < 0.001 vs. CTR); (**b**) Dose-dependent effect of *Fragaria-*derived EPDENs (µg/mL) on H_2_O_2_ (400 µM)-induced cytotoxicity (*n* = 4) (*** *p* < 0.001).

**Figure 4:**
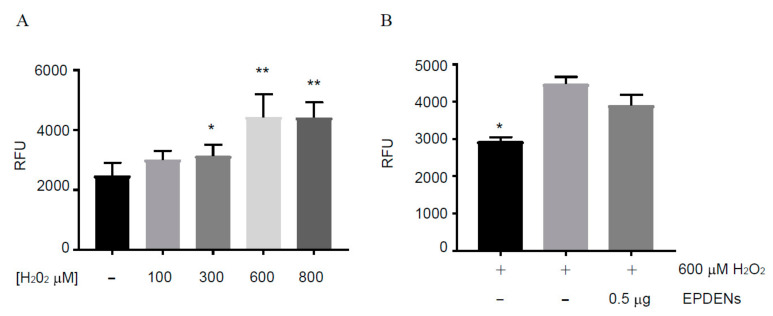
Effect of Fragaria-derived EPDENs on ROS production in human ADMSC. (**A**) Dose-dependent effect of H_2_O_2_ on ROS production (* *p* < 0.05 vs. CTR and ** *p* < 0.01 vs. CTR); (**B**) Effect of Fragaria-derived EPDENs (0.5 µg/mL) on H_2_O_2_ (600 µM)-induced ROS production (* *p* < 0.05 vs. H_2_O_2_).

As shown in Figure 3a, treatment with H_2_O_2_ for 24 h reduced cell viability in a concentration-dependent manner. We identified 400 μM H_2_O_2_ as the concentration that significantly decreased cell viability by approximately 50%. Therefore, we used this concentration to induce ADMSC oxidative stress in subsequent experiments. To evaluate the protective effect of *Fragaria*-derived EPDENs, ADMSCs were pretreated for 24 h with increasing concentrations of EPDENs before the addition of H_2_O_2_ for 24 h. Cell viability results indicated that *Fragaria*-derived EPDENs improved ADMSC survival in a dose-dependent manner, with a significant increase at the 1 and 2 μg/mL doses (*p* < 0.001 untreated cells (CTR) vs. H_2_O_2_, *p* < 0.001 1 and 2 μg/mL vs. H_2_O_2_) (Figure 3b). Intracellular ROS production was evaluated in ADMSCs pretreated with *Fragaria*-derived EPDENs. A slight decrease in ROS production was detected after the pretreatment with 0.5 μg/mL EPDENs (Figure 4).

### 3.6. Content of Small RNAs in Fragaria-Derived EPDENs

**Figure 5:**
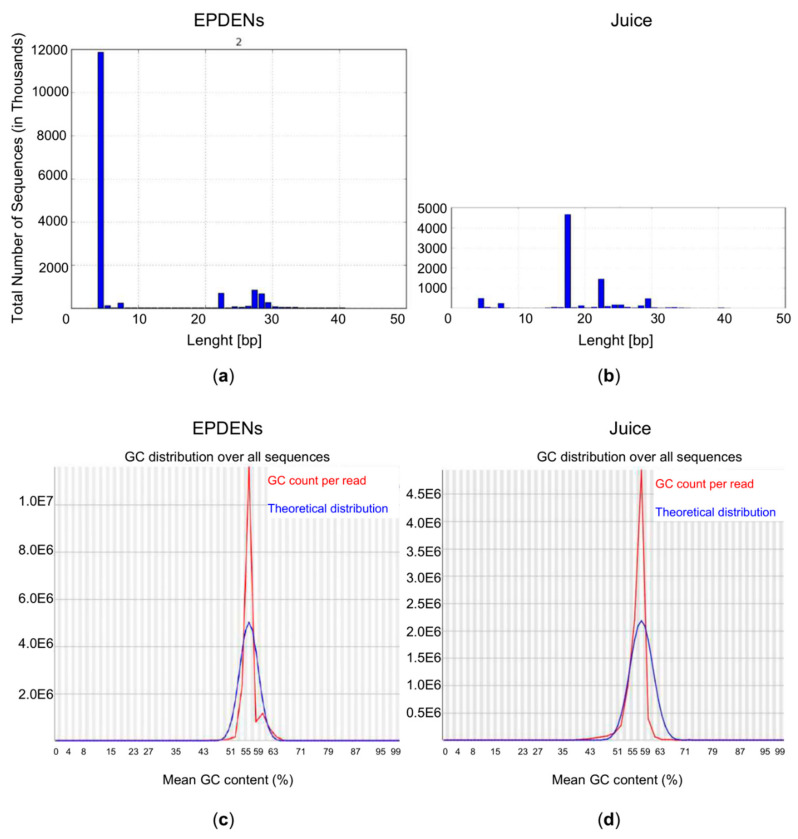
Size distribution of small RNAs of (**a**) *Fragaria*-derived EPDENs with respect to (**b**) *Fragaria* whole juice. GC frequency of sequences of (**c**) *Fragaria*-derived EPDENs with respect to (**d**) *Fragaria* whole juice.

Small RNAs isolated from *Fragaria* EPDENs or whole juice show a very different size distribution pattern. We identified small RNAs in EPDENs with a specific length size distribution that was distinguishable from that of the whole juice from which they were derived. Our results showed a selective enrichment of four nucleotide small RNAs and a minor class of 23–29 nucleotide small RNAs in EPDENs. The majority of the small RNAs derived from *Fragaria* whole juice ranged in size between 17 and 30 nucleotides in length (Figure 5).

### 3.7. Identification of miRNAs in Fragaria-Derived EPDENs

To investigate whether *Fragaria-*derived nanovesicles also contain miRNAs, we isolated RNA from nanovesicles and compared the miRNAs sequences found in *Fragaria* EPDENs with its juice and the miRNA library of *Arabidopsis thaliana*. The comparison of the identified sequences in *Fragaria*-derived EPDENs with the miRNA library of *Arabidopsis thaliana* (427 miRNA) permitted the identification of different miRNAs in the whole juice from *Fragaria* and only one miRNA in EPDENs (Table 2). In fact, miR166g was found both in EPDENs and in the whole juice, although in a lower concentration (lower number of reads).

## 4. Discussion

Dietary intake of fruits and vegetables has consistently emerged as an effective strategy to promote human health. Consumption of fruits and vegetables is associated with a reduced risk of chronic diseases, such as cardiovascular disease, diabetes, cancer, and age-related functional decline [23,24,25]. Brondani et al. [26] showed that dietary patterns rich in fruits and vegetables are associated with a reduced risk of bone fractures and the prevention of osteoporotic fractures. However, the mechanisms through which plant-rich diets achieve such effects are still unclear. Bioactive components present in plants, such as flavonoids, phenolic acids, and carotenoids, seem to play an important role in lowering the risks of major chronic diseases [27]. However, there is an intriguing phenomenon in which the synthetic supplements of phytochemicals are often not as efficient as complex plant materials, such as vitamins. This may imply the presence of diverse bioactive substances in plants and the existence of certain unidentified bioactive components. Interestingly, recent studies have shown the presence of exosome-like nanoparticles in different plants and fruits as well as the ability of these nanoparticles to carry bioactive compounds [17,28,29]. Exosome-like nanoparticles involved in plant cell–cell communication could potentially regulate the innate immunity of plants and can also transport mRNAs, miRNAs, bioactive lipids, and proteins to recipient cells in different contexts [30]. Ju et al. [13] reported that exosome-like nanoparticles can penetrate the intestinal mucus barrier and can be taken up by mouse intestinal stem cells. Furthermore, in some fruits, such as lemons and blueberry fruit, cross-kingdom biological effects have been identified, suggesting new possible applications for fruit-derived nanovesicles [12,31].

In this study, we reported, for the first time, the isolation, purification, and characterization of EPDENs from strawberry fruits of *Fragaria* x *ananassa* (cv. Romina). Strawberries may be classified as functional foods because they are rich sources of phytochemicals and vitamins with well-known health benefits [32,33]. Romina is recognized for its high content of micronutrients and bioactive compounds, which confer interesting biological activities to the fruits [19]. Their antioxidant capacity and hypolipidemic and antiatherosclerotic effects, as well as their antitumor activities, have been highlighted in our recent papers by using whole Romina extracts or its anthocyanin fraction [34,35,36,37].

Here, we demonstrated that Romina fruits contain a homogeneous population of nanovesicles, with a dimension and structure attributable to exosome-like nanoparticles [11]. *Fragaria*-derived EPDENs show size and morphology similar to those of EVs from mammalian cells and EV-derived plant cells [12], even if their size distribution showed a higher percentage of smaller vesicles in EPDENs compared to EVs samples. Interestingly, *Fragaria*-derived EPDENs are internalized by human MSC and do not exert any significant toxicity on cells.

Given the significant content of Romina fruit of vitamin C, folic acid, and flavonols combined with a high content of anthocyanin and an elevated antioxidant capacity [21], we hypothesized that EPDENs released from Romina may contain molecules associated with oxidative stress. Our results revealed that *Fragaria*-derived EPDENs are able to protect the response of human MSCs to oxidative stress. Interestingly, analysis of EPDENs cargo revealed the presence of a high content of vitamin C (0.416 nmoles vitamin C/µg EPDENs). We previously found that *Citrus limon* L.-derived EPDENs also contain vitamin C but in lower amounts (0.009 nmoles vitamin C/µg EPDENs) [12]. This might explain the higher efficacy of *Fragaria*-derived EPDENs to prevent oxidative stress in human cells when compared with *Citrus limon* L.-derived EPDENs.

Vitamin C, also known as ascorbic acid, is a natural free radical scavenger that protects plant cells from oxidative stress [38]. Vitamin C is an essential nutrient for humans and a cofactor in different enzymatic reactions, participating in a variety of biological functions, both in mammals and plants [39].

In the human diet, ascorbic acid has gained popularity as an antioxidant for its capacity to counteract the production of free radicals in cells and as a natural chemopreventive for the reduction of risk factors for cardiovascular diseases and cancer [38,40]. Our results identified a new emerging role for *Fragaria*-derived EPDENs as transporters of vitamin C and their antioxidant capacity. Oxidative damage has many pathological implications in human health, and, in this respect, the beneficial effects of food intake of vitamin C have important implications in aging, bone health, cardiovascular disease, neurodegenerative diseases, and cancer [41,42,43]. It is thus important to consider these plant-derived nanovesicles as new components of our food since nanovesicles can preserve antioxidant properties longer than free antioxidant substances. Furthermore, EVs are able to resist gastric pepsin solutions and intestinal pancreatic and bile extract solutions [10]. The untimely degradation and the lack of intestinal absorption of vitamin C might thus be overcome by its loading in extracellular vesicles to be used as a safe dietary supplement or as a therapeutic agent. Collectively, these characteristics of EPDENs could promote their use in clinical strategies or support traditional therapies. However, further studies are required to understand the mechanisms and factors underlying the efficacy of strawberry biocompounds.

The analysis of *Fragaria*-derived EPDENs cargo also revealed the presence of small RNAs encapsulated in the nanovesicles. Small RNAs play essential roles in the regulation of plant development and immunity during abiotic and biotic stress responses [44]. The characteristic 2′-O-methylation of plant miRNAs enhances their stability in harsh environments and might protect them from enzymatic digestion [45]. Moreover, plant miRNAs have been found to be associated with RNA-binding proteins or enveloped in nano and microvesicles, which, additionally, protect them from degradation [46,47]. Baldrich et al. [47] demonstrated that specific miRNAs and siRNAs are preferentially loaded into plant EVs. This selective loading into extracellular vesicles similarly occurs in mammal-derived EVs [48]. Accordingly, we found a specific enrichment of miR166g in *Fragaria-*derived EPDENs. Specific activities were identified for miR166g in plants. In *Arabidopsis* jabba-1D, the overexpression of miR166g causes morphological defects in shoot apical meristems and stem vasculature, disrupting the morphogenesis of leaves [49]. Leaf curvature was also affected by miR166g in *Brassica rapa* [50].

However, the possibility of cross-kingdom miRNA activity is still highly debatable. Zhang et al. [51] identified exogenous miRNAs both in human and animal sera, but this finding has not been confirmed by subsequent studies [52,53], and several doubts related to the biological relevance of the reported copy number of individual sequences have been raised. Conversely, Link et al. [53] found detectable levels of plant miR-168 in human feces, normal gastric, and colon cancer mucosa, suggesting potential interspecies activity. Interestingly, a therapeutic effect of plant miRNAs in the prevention of chronic inflammation has been shown in a mouse model of human multiple sclerosis. Unexpectedly, the biological activity of plant-derived miRNA was exerted with a sequence-independent mechanism [54].

A better understanding of the possible cross-talk between exogenous miRNA and human pathophysiological conditions will open new opportunities for the development of plant-based nutraceutical approaches.

## 5. Conclusions

In this study, we isolated, purified, and characterized exosome-like nanoparticles from *Fragaria x ananassa* (cv. Romina) juice. These EPDENs can be internalized by human cells, without exerting toxic effects. *Fragaria-*derived EPDENs prevent oxidative stress on human cells, possibly through the activity of vitamin C, which we found preserved in their cargo. Moreover, *Fragaria*-derived EPDENs carry small RNA, and potential activity on human cells will open new nutraceutical approaches and enable discussions on the possibility of cross-kingdom miRNA activity.

## Figures and Tables

**Table 1:** Nanovesicles size distribution of *Fragaria* x *ananassa*, *Citrus limon* L., and ADMSCs.

	Nanovesicles Size			
	30–49 nm	50–100 nm	101–121 nm	122–191 nm
*Fragaria x ananassa* EPDENs	58.8%	35.6%	3%	2.6%
*Citrus limon* L. EPDENs	41.2%	45.5%	9%	4.3%
ADMSC EVs	3%	44%	35.3%	17.7%

**Table 2:** miRNAs that were identified in *Fragaria*-derived EPDENs after a comparison with the miRNA library of *Arabidopsis thaliana*. The mapping of the number of reads against each miRBase is shown (only exact matches were allowed).

miRNA Name (Accession Number)	*Fragaria*-Derived EPDENs (*n*. of Reads)	*Fragaria* Juice (*n*. of Reads)
miR166g (MIMAT0000195)	11	37
miR168b-5p (MIMAT0000199)	0	25
miR396a-5p (MIMAT0000944)	0	4
miR159b-3p (MIMAT0000207)	0	4
miRNA159a (MIMAT0000177)	0	14

## Data Availability

RNA seq raw data are available on request from the corresponding author.

## References

[1] Raposo G., Stoorvogel W. (2013). Extracellular vesicles: Exosomes, microvesicles, and friends. J. Cell Biol..

[2] Kalra H., Drummen G.P., Mathivan S. (2016). Focus on Extracellular Vesicles: Introducing the Next Small Big Thing. Int. J. Mol. Sci..

[3] Lopez-Verrilli M.A., Court F.A. (2013). Exosomes: Mediators of communication in eukaryotes. Biol. Res..

[4] Perut F., Roncuzzi L., Baldini N. (2019). The emerging role of extracellular vesicles in osteosarcoma. Front. Oncol..

[5] Schwab A., Meyering S.S., Lepene B., Iordanskiy S., van Hoek M.L., Hakami R.M., Kashanchi F. (2015). Extracellular vesicles from infected cells: Potential for direct pathogenesis. Front. Microbiol..

[6] De Toledo Martins S., Szwarc P., Goldenberg S., Alves L.R. (2019). Extracellular vesicles in fungi: Composition and functions. Curr. Top. Microbiol. Immunol..

[7] Woith E., Fuhrmann G., Melzig M.F. (2019). Extracellular Vesicles—Connecting Kingdoms. Int. J. Mol. Sci..

[8] Liu B., Lu Y., Chen X., Muthuraj P.G., Li X., Pattabiraman M., Zempleni J., Kachman S.D., Natarajan S.K., Yu J. (2020). Protective Role of Shiitake Mushroom-Derived Exosome-Like Nanoparticles in D-Galactosamine and Lipopolysaccharide-Induced Acute Liver Injury in Mice. Nutrients.

[9] Regente M., Pinedo M., Elizalde M., de la Canal L. (2012). Apoplastic exosome like vesicles: A new way of protein secretion in plants?. Plant Signal. Behav..

[10] Mu J., Zhuang X., Wang Q., Jiang H., Deng Z.B., Wang B., Zhang L., Kakar S., Jun Y., Miller D. (2014). Interspecies communication between plant and mouse gut host cells through edible plant derived exosome-like nanoparticles. Mol. Nutr. Food Res..

[11] Zhang M., Viennois E., Xu C., Merlin D. (2016). Plant derived edible nanoparticles as a new therapeutic approach against diseases. Tissue Barriers.

[12] Baldini N., Torreggiani E., Roncuzzi L., Perut F., Zini N., Avnet S. (2018). Exosome-like nanovesicles isolated from Citrus limon L. have anti-oxidative effect. Curr. Pharm. Biotechnol..

[13] Ju S., Mu J., Dokland T., Zhuang X., Wang Q., Jiang H., Xiang X., Deng Z.B., Wang B., Zhang L. (2013). Grape exosome-like nanoparticles induce intestinal stem cells and protect mice from DSS-induced colitis. Mol. Ther..

[14] Raimondo S., Naselli F., Fontana S., Monteleone F., Lo Dico A., Saieva L., Zito G., Flugy A., Manno M., Di Bella M.A. (2015). Citrus limon-derived nanovesicles inhibit cancer cell proliferation and suppress CML xenograft growth by inducing TRAIL-mediated cell death. Oncotarget.

[15] Wang Q.L., Ren Y., Mu J.Y., Egilmez N.K., Zhuang X.Y., Deng Z.B., Zhang L.F., Yan J., Miller D., Zhang H.G. (2015). Grapefruit Derived Nanovectors Use an Activated Leukocyte Trafficking Pathway to Deliver Therapeutic Agents to Inflammatory Tumor Sites. Cancer Res..

[16] Zhuang X., Deng Z.B., Mu J., Zhang L., Yan J., Miller D., Feng W., McClain C.J., Zhang H.G. (2015). Ginger-derived nanoparticles protect against alcohol-induced liver damage. J. Extracell. Vesicles.

[17] Pérez-Bermúdez P., Blesa J., Soriano J.M., Marcilla A. (2017). Extracellular vesicles in food: Experimental evidence of their secretion in grapefruits. Eur. J. Pharm. Sci..

[18] Cianciosi D., Simal-Gandara J., Forbes-Hernández T.Y. (2019). The importance of berries in the human diet. Mediterr. J. Nutr. Metab..

[19] Mezzetti B., Giampieri F., Zhang Y.T., Zhong C.F. (2018). Status of strawberry breeding programs and cultivation systems in Europe and the rest of the world. J. Berry Res..

[20] Forbes-Hernández T.Y., Gasparrini M., Afrin S., Cianciosi D., González-Paramás A.M., Santos-Buelga C., Mezzetti B., Quiles J.L., Battino M., Giampieri F.V. (2017). Strawberry (cv. Romina) Methanolic Extract and Anthocyanin-Enriched Fraction Improve Lipid Profile and Antioxidant Status in HepG2 Cells. Int. J. Mol. Sci..

[21] Capocasa F., Balducci F., Di Vittori L., Mazzoni M., Stewart D., Williams S., Hargreaves R., Bernardini D., Danesi L., Zhong C.F. (2016). Romina and Cristina: Two New Strawberry Cultivars with High Sensorial and Nutritional Values. Int. J. Fruit Sci..

[22] Perut F., Roncuzzi L., Zini N., Massa A., Baldini N. (2019). Extracellular Nanovesicles Secreted by Human Osteosarcoma Cells Promote Angiogenesis. Cancers.

[23] Boeing H., Bechthold A., Bub A., Ellinger S., Haller D., Kroke A., Leschik-Bonnet E., Müller M.J., Oberritter H., Schulze M. (2012). Critical review: Vegetables and fruit in the prevention of chronic diseases. Eur. J. Nutr..

[24] Battino M., Forbes-Hernández T.Y., Gasparrini M., Afrin S., Cianciosi D., Zhang J., Manna P.P., Reboredo-Rodríguez P., Varela Lopez A., Quiles J.L. (2019). Relevance of functional foods in the Mediterranean diet: The role of olive oil, berries and honey in the prevention of cancer and cardiovascular diseases. Crit. Rev. Food Sci. Nutr..

[25] Aune D., Giovannucci E., Boffetta P., Fadnes L.T., Keum N., Norat T., Greenwood D.C., Riboli E., Vatten L.J., Tonstad S. (2017). Fruit and vegetable intake and the risk of cardiovascular disease, total cancer and all-cause mortality-a systematic review and dose-response meta-analysis of prospective studies. Int. J. Epidemiol..

[26] Brondani J.E., Comim F.V., Flores L.M., Martini L.A., Premaor M.O. (2019). Fruit and vegetable intake and bones: A systematic review and meta-analysis. PLoS ONE.

[27] Liu R.H. (2013). Health-promoting components of fruits and vegetables in the diet. Adv. Nutr..

[28] Sagini K., Urbanelli L., Buratta S., Leonardi L., Emiliani C. (2017). Nanovesicles from plants as edible carriers of bioactive compounds. Agrolife Sci. J..

[29] Akuma P., Okagu O.D., Udenigwe C.C. (2019). Naturally occurring exosome vesicles as potential delivery vehicle for bioactive compounds. Front. Sustain. Food Syst..

[30] Iravani S., Varma R.S. (2019). Plant-derived edible nanoparticles, and miRNAs: Emerging frontier for therapeutics and targeted drug-delivery. ACS Sustain. Chem. Eng..

[31] De Robertis M., Sarra A., D’Oria V., Mura F., Bordi F., Postorino P., Fratantonio D. (2020). Blueberry-Derived Exosome-Like Nanoparticles Counter the Response to TNF-α-Induced Change on Gene Expression in EA. hy926 Cells. Biomolecules.

[32] Mazzoni L., Perez-Lopez P., Giampieri F., Alvarez-Suarez J.M., Gasparrini M., Forbes-Hernandez T.Y., Quiles J.L., Mezzetti B., Battino M. (2016). The genetic aspects of berries: From field to health. J. Sci. Food Agric..

[33] Basu A., Nguyen A., Betts N.M., Lyons T.J. (2014). Strawberry as a functional food: An evidence-based review. Crit. Rev. Food Sci. Nutr..

[34] Forbes-Hernández T.Y., Cianciosi D., Ansary J., Mezzetti B., Bompadre S., Quiles J.L., Giampieri F., Battino M. (2020). Strawberry (*Fragaria x ananassa* cv. Romina) methanolic extract promotes browning in 3T3-L1 cells. Food Funct..

[35] Forbes-Hernández T.Y., Giampieri F., Gasparrini M., Afrin S., Mazzoni L., Cordero M.D., Mezzetti B., Quiles J.L., Battino M. (2017). Lipid Accumulation in HepG2 Cells Is Attenuated by Strawberry Extract through AMPK Activation. Nutrients.

[36] Forbes-Hernández T.Y., Afrin S., Cianciosi D., Manna P.P., Zhang J., Gasparrini M., Reboredo-Rodríguez P. (2018). Strawberry extract attenuates oxidative stress in 3T3-L1 cells. J. Berry Res..

[37] Giampieri F., Islam M.S., Greco S., Gasparrini M., Forbes Hernandez T.Y., Delli Carpini G., Giannubilo S.R., Ciavattini A., Mezzetti B., Mazzoni L. (2019). Romina: A powerful strawberry with in vitro efficacy against uterine leiomyoma cells. J. Cell. Physiol..

[38] Paciolla C., Fortunato S., Dipierro N., Paradiso A., De Leonardis S., Mastropasqua L., de Pinto M.C. (2019). Vitamin C in plants: From functions to biofortification. Antioxidants.

[39] Chisnall M., Macknight R., Hossain M., Munné-Bosch S., Burritt D., Diaz-Vivancos P., Fujita M., Lorence A. (2017). Importance of Vitamin C in Human Health and Disease. Ascorbic Acid in Plant Growth, Development and Stress Tolerance.

[40] Sunil Kumar B.V., Singh S., Verma R. (2017). Anticancer potential of dietary vitamin D and ascorbic acid: A review. Crit. Rev. Food Sci. Nutr..

[41] Granger M., Eck P. (2018). Dietary Vitamin C in Human Health. Adv. Food Nutr. Res..

[42] Chin K.Y., Ima-Nirwana S. (2018). Vitamin C and Bone Health: Evidence from Cell, Animal and Human Studies. Curr. Drug Targets.

[43] Blaszczak W., Barczak W., Masternak J., Kopczyński P., Zhitkovich A., Rubiś B. (2019). Vitamin C as a Modulator of the Response to Cancer Therapy. Molecules.

[44] Zhu C., Liu T., Chang Y.N., Duan C.G. (2019). Small RNA Functions as a Trafficking Effector in Plant Immunity. Int. J. Mol. Sci..

[45] Xie W., Melzig M.F. (2018). The Stability of Medicinal Plant microRNAs in the Herb Preparation Process. Molecules.

[46] Zhao Y., Mo B., Chen X. (2012). Mechanisms That Impact microRNA Stability in Plants. RNA Biol..

[47] Baldrich P., Rutter B.D., Karimi H.Z., Podicheti R., Meyers B.C., Innes R.W. (2019). Plant Extracellular Vesicles Contain Diverse Small RNA Species and Are Enriched in 10- to 17-Nucleotide “Tiny” RNAs. Plant Cell..

[48] Baglio S.R., Rooijers K., Koppers-Lalic D., Verweij F.J., Pérez Lanzón M., Zini N., Naaijkens B., Perut F., Niessen H.W., Baldini N. (2015). Human bone marrow- and adipose-mesenchymal stem cells secrete exosomes enriched in distinctive miRNA and tRNA species. Stem Cell Res. Ther..

[49] Williams L., Grigg S.P., Xie M., Christensen S., Fletcher J.C. (2005). Regulation of Arabidopsis shoot apical meristem and lateral organ formation by microRNA miR166g and its AtHD-ZIP target genes. Development.

[50] Ren W., Wang H., Bai J., Wu F., He Y. (2018). Association of microRNAs with Types of Leaf Curvature in Brassica rapa. Front. Plant Sci..

[51] Zhang L., Hou D., Chen X., Li D., Zhu L., Zhang Y., Li J., Bian Z., Liang X., Cai X. (2012). Exogenous plant MIR168a specifically targets mammalian LDLRAP1: Evidence of cross-kingdom regulation by microRNA. Cell Res..

[52] Dickinsons B., Zhang Y., Petrick Y.S., Heck G., Ivashuta S., Marshall W.S. (2013). Lack of Detectable Oral Bioavailability of Plant MicroRNAs After Feeding in Mice. Nat. Biotechnol..

[53] Link J., Thon C., Schanze D., Steponaitiene R., Kupcinskas J., Zenker M., Canbay A., Malfertheiner P., Link A. (2019). Food-Derived Xeno-microRNAs: Influence of Diet and Detectability in Gastrointestinal Tract-Proof-of-Principle Study. Mol. Nutr. Food Res..

[54] Cavalieri D., Rizzetto L., Tocci N., Rivero D., Asquini E., Si-Ammour A., Bonechi E., Ballerini C., Viola R. (2016). Plant microRNAs as novel immunomodulatory agents. Sci. Rep..

